# Dredging Intensity: A Spatio-Temporal Indicator for Managing Marine Resources

**DOI:** 10.1007/s00267-018-1084-8

**Published:** 2018-07-24

**Authors:** Henry Bokuniewicz, Sung Gheel Jang

**Affiliations:** 1 School of Marine and Atmospheric Sciences, Stony Brook University, Stony Brook, NY 11794-5000 USA; 2 The Geospatial Center, Sustainability Studies Program, Stony Brook, USA

**Keywords:** Beach nourishment, Dredging, Marine sand management

## Abstract

The sustainability of offshore sand reserves and the impact of their exploitation for coastal resilience can be assessed by resource managers *via* GIS. The GIS model to do this requires monitoring of the dredger location (including speed and displacement, if available). The designated borrow area is divided into grid cells, in this example, 100 × 100 m. The aggregate count of positions in each cell can be displayed in a graphic image called a “heat map” (or “density map” or “timeprint”) where various intensities of colors represents the number vessel locations in each designated cell over the entire time period of interest as a surrogate for dredging intensity. Because sand dredging using a trialing hopper dredge is done at slow speeds, the aggregate time that a dredger spends in each cell can be modified by dredger speed to discriminate time spent actually removing sand from time spent in transit. If vessel displacements is also monitored, increases in displacement will also identify times and locations of active extraction. In this way, areas of disturbed benthic habitat can be identified, even if changes in bathymetry are not resolved.

## Introduction

Coastal communities increasingly are challenged by the need to exploit the offshore sand resources in order to recover from beach erosion brought on by severe storms. On the U.S. east coast, recent extreme weather, like Hurricane Katrina and “Superstorm” Sandy”, brought such concerns to the forefront. Federal agencies including the U.S. Army Corps of Engineers (USACE), Bureau of Ocean Energy Management (BOEM), and State resource agencies are faced with the management of diverse marine resources to sustain the ability to continue to address community vulnerability in the face of major disasters. The sustainability of offshore sand resources depends on three conditions (Hilton 1994). First, the volume removed by dredging should be insignificant compared with the total volume of the resource, or, second, dredging should occur at a rate that is commensurate the rate of natural recovery of the resource. Third, the adverse impacts on habitats should be minimized so that ecosystem goods and services can be sustained. The three conditions require different measures of dredging intensity. In this article, we discuss a GIS approach to track the extraction of offshore sand resources specifically to better assess adverse impacts on benthic habitats.

Offshore habitat-mapping has come to rely on the use of abiotic proxies to provide high-resolution habitat maps to resource managers (Brown and Blondel 2009; Buhl-Mortensen et al. 2015). Multibeam echo sounders in particular have revolutionized mapping of benthic habitats (Brown and Blondel 2009), because their products can discriminate differences in habitat value using bathymetric information, such as slope, and topographic roughness, resolved to one-square-meter areas over large parts of the sea floor (Calvert et al. 2015). Discrimination of habitats over distances of as little as 200 m has been found to capture important changes over distances of tens of meters among the clutter of very small-scale variation (Calvert et al. 2015). As a result, the ability to map bottom disturbances caused by dredging on this scale would be useful.

Ocean-going trailing suction hopper dredges are often used to excavate marine sand for beach restoration. These are self-propelled vessels that have two long suction pipes, called dragarms, attached, one on each side. The mouths of the dragarms, called “dragheads” are dragged over the seabed to extract sand. The other end is attached through pumps to the ship’s hopper. Offshore dredging is undertaken in designated “borrow” areas. When the dredge arrives at the designated borrow area, its speed is reduced to two or three knots (Vlasblom 2007 p13). The suction pipes’ dragheads are lowered to the sea floor. Sand is pumped through the dragheads and suction pipes into the ship’s hopper, later to be transported to the beach nourishment site and discharged. Trailer-suction dredging results in tracks across the sea floor that is usually less than 0.55 m deep (van Moorsel and Waardenburg 1990; Kenny and Rees 1994; Boyd et al. 2003; Davies and Hitchcock 1992). Removal of the surface 0.5 m of the seabed is sufficient to eliminate the benthos from the deposits. So, as will be discussed later, some habitats can be impacted in places where the changes in bathymetry might be unresolved by bathymetric surveys.

Dredging intensity has been defined as volume extracted/area/time (ICES 2014), but “volume” can be an elusive parameter. Some projects had been documented as permitted volumes or barge-loads, while others use in-place volumes and all have inherent uncertainties. For contracting purposes, volumes extracted are often verified by pre-and-post-project surveys at the site of placement or at the borrow site. Bathymetric surveys include an inherent uncertainty both in recorded water depth and the ship’s location. Errors can be 0.15 or 0.25 m (e.g., Wijnberg and Terwindt 1995). An error of 0.05 m in depth alone in a survey covering 2.5 km of shoreline across a nearshore width of 40 m amounts to an uncertainty of 50,000 m^3^ (Gibeaut et al. 1998). Uncertainties of this magnitude, however, may be tolerable in project that might involve a million cubic meters or more.

Where extractions are concentrated in deep pits over small areas, such uncertainties also can be negligible in the calculation of pre-volumes and post-volumes. In many instances, however, deep pits are avoided intentionally to prevent adverse impacts on wave conditions or water column stratification and, instead, sand is removed in thin layers over larger areas. Because of the resolution of the surveys, disturbance of the seafloor involving the extraction of thin layers over large areas around the margins, and the habitats in those areas, may go undocumented by conventional bathymetric comparisons.

Bathymetric surveys aside, modern, electronic monitoring systems (EMSs) are capable of recording real-time data on dredging operations such as location, ship speed, depth of cut, and sediment mixture concentration to name a few (Francingues et al. 2000). In the U.S., monitoring systems are used for federal projects within National Dredging Quality Management (DQM) Program of the USACE. The DQM program collects real-time information every six seconds recording the date, time, position, speed, vessel draft, dragarm depths, density of the pumped slurry, and pumping rate then use these data to calculate displacement, hopper volume, ullage (the amount by which the hopper falls short of being full), draghead position, and the depth of the cut (http://dqm.usace.army.mil/, accessed December 2017). The USACE’s Mobile District maintains DQM databases for federal beach nourishment, coastal restoration, and navigation projects. Although some of these data are proprietary, official users might obtain basic nonproprietary information from the DQM support team. The record of displacement is particularly useful because, when dredging in the borrow area, the displacement of the vessel will increase for one reading to the next. However, complete data such as this is not collected everywhere and, even when it is collected, can be effectively unavailable to resource managers because of its proprietary classification.

In principle, total extracted volume can be calculated from the DQM data, but evaluating volume extracted/area/time can be very difficult, and even impossible for many countries (ICES 2015). As discussed, excavated volumes are determined routinely by bathymetric surveys instead. For assessment of habitat impact, the data to calculate aggregate time a dredger spends in designated borrow site might be used as a surrogate for volume/area/time. Not only are measurements of time/area likely to be more widely available, but also they are capable of locating areas of habitat disturbance that may be undetected in bathymetric surveys because of uncertainties in the measured water depth. It should be kept in mind, however, that dredging time per area does not take into account other important parameters, such as the size of the dredging vessel, the type of material extracted, and whether screening takes place or not.

In the absence of DQM data, the automatic identification system (AIS) vessel tracking data might be used to assess the intensity of dredging activity in specific areas. AIS is capable of providing a vessel’s longitude and latitude, in addition to its course, heading and speed, routinely aggregated at five-minute intervals. AIS positioning data for a particular vessel in the United States and time period can be assembled by request by the U.S. Coast Guard.

**Fig. 1:**
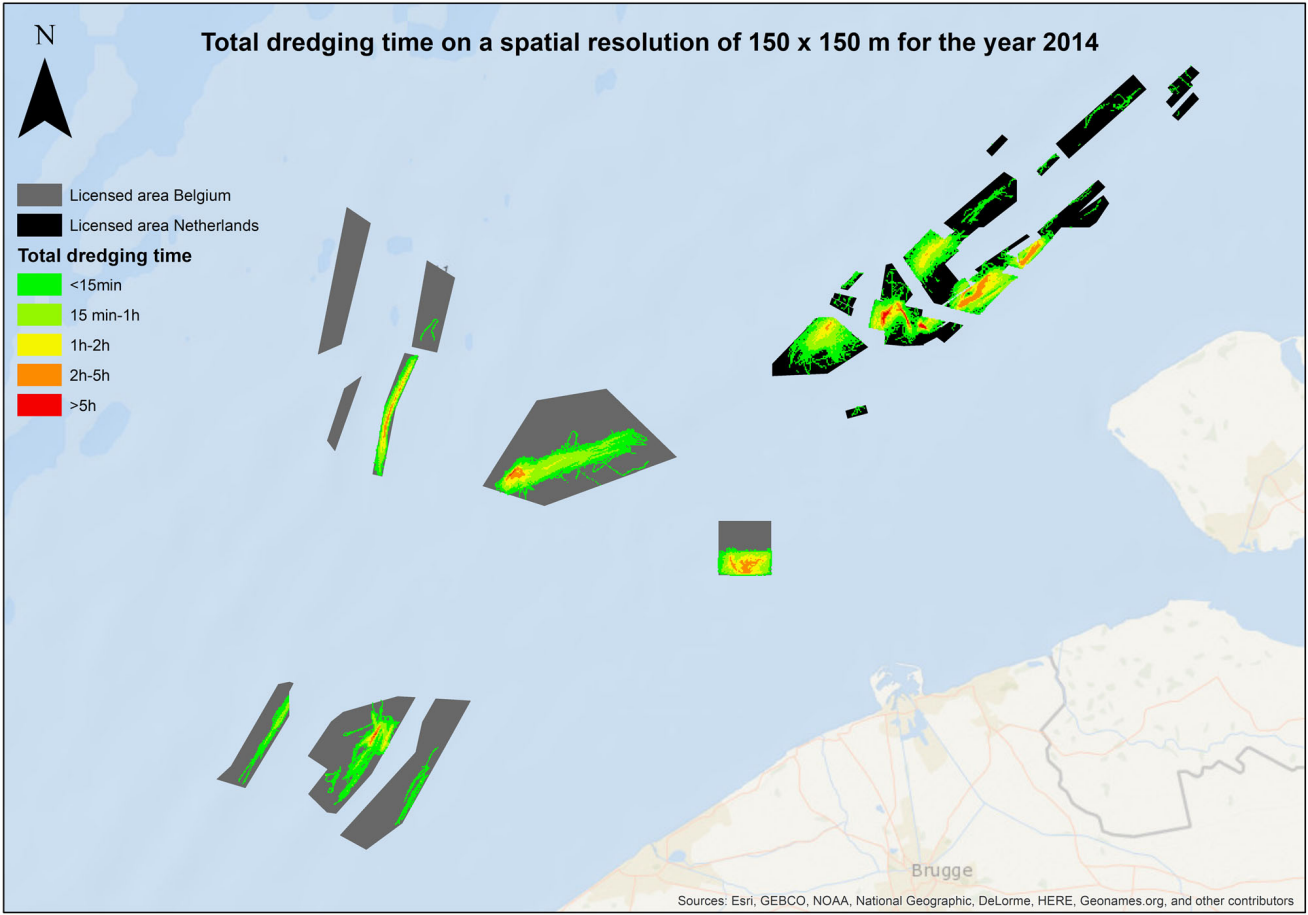
Dredging pressure at Belgium extraction sites at a resolution of 50 × 50 m for the 2014 (De Backer et al. 2017)

The GIS application described here can provide a method of tracking the use of designated borrow areas in order to maintain the ability to manage offshore sand resources in a coordinated and sustainable manner. Results can be displayed in a graphic image called a “heat map”, or “density map”, or “timeprint”, where various intensities of colors represents the number vessel locations in each designated cell over the entire time period of interest (https://www.navcen.uscg.gov/?pageName=NAISDataFormats, accessed December, 2017). “Time” could be reported either as the total number of hours or minutes dredged over the course of a year. In the United States, because most large beach renourishment projects are done by the USACE, the federal fiscal year may be most appropriate. A test case had been run by the Working Group on the Effects of Extraction of Marine Sediments on Marine Ecosystems of the International Council for the Exploration of the Sea (ICES 2016). Using both Belgian EMS data and the commercial EMS data of the Netherlands, output maps were generated showing the total time dredged, a “timeclock-print”, in an area of 50 × 50 m over the course of the year 2014 (De Backer et al. 2017; Fig. 1).

## Methods

The AIS or EMS data over many projects and time frames can be processed in GIS to identify dredging intensity. Data used in this illustration were provided through the Bureau of Ocean Energy Management (BOEM) by the Navigation Data Center of the USACE, Institute for Water Resources. Data were reported every 5 min identifying a load number, date and time, vessel longitude, vessel latitude, vessel speed in knots, vessel heading, vessel course, forward vessel draft, aft vessel draft and displacement in long tons (1016 kg). The data used for the examples here were DMP data from an actual dredging operation over a six-month period; however, for this illustration, the geographical coordinates, vessel identification and calendar dates were removed. A hypothetical borrow area was designated for the purpose of this example. As described in the following procedure, the amount of time associated with every point location is summed into a single cell value.

In order to create a raster dataset in which the values of each cell represent the total amount of time that vessels have spent in a predefined cell (100 × 100 m), various steps of data manipulation and data conversion were performed using ArcGIS 10.5.1 as follows:

1. A “vessel positions” feature class in the file geodatabase was created using the “From *X*-*Y* Table” option in ArcGIS because the 5-min interval vessel point data in comma separate value contains the vessel longitude (*X*) and the vessel latitude (*Y*). The NAD 1983 geographic coordinate system was selected as the input coordinate system of the vessel point data.
2. Because the time density needs to be summarized by metric cell (100 × 100 m), the vessel point data in the NAD 1983 geographic coordinate system was projected to the WGS 1984 Web Mercator Auxiliary Sphere projected coordinate system.
3. A new integer field, called VALUE, was added to the attribute table of the vessel point data to store the 5-min time interval. Using Field Calculator, all cells in the VALUE field were populated with the numeric value 5.
4. To convert the vessel point data to raster, the Point to Raster tool was used. Parameters used for this conversion are as follows: the VALUE field of the point data as value field of output raster, SUM as cell assignment type, and 100 as cell size. The cell assignment type makes the Point to Raster tool compute total time per each cell by summing up the numeric values of the VALUE field of point features within each cell (100 × 100 m).

**Fig. 2:**
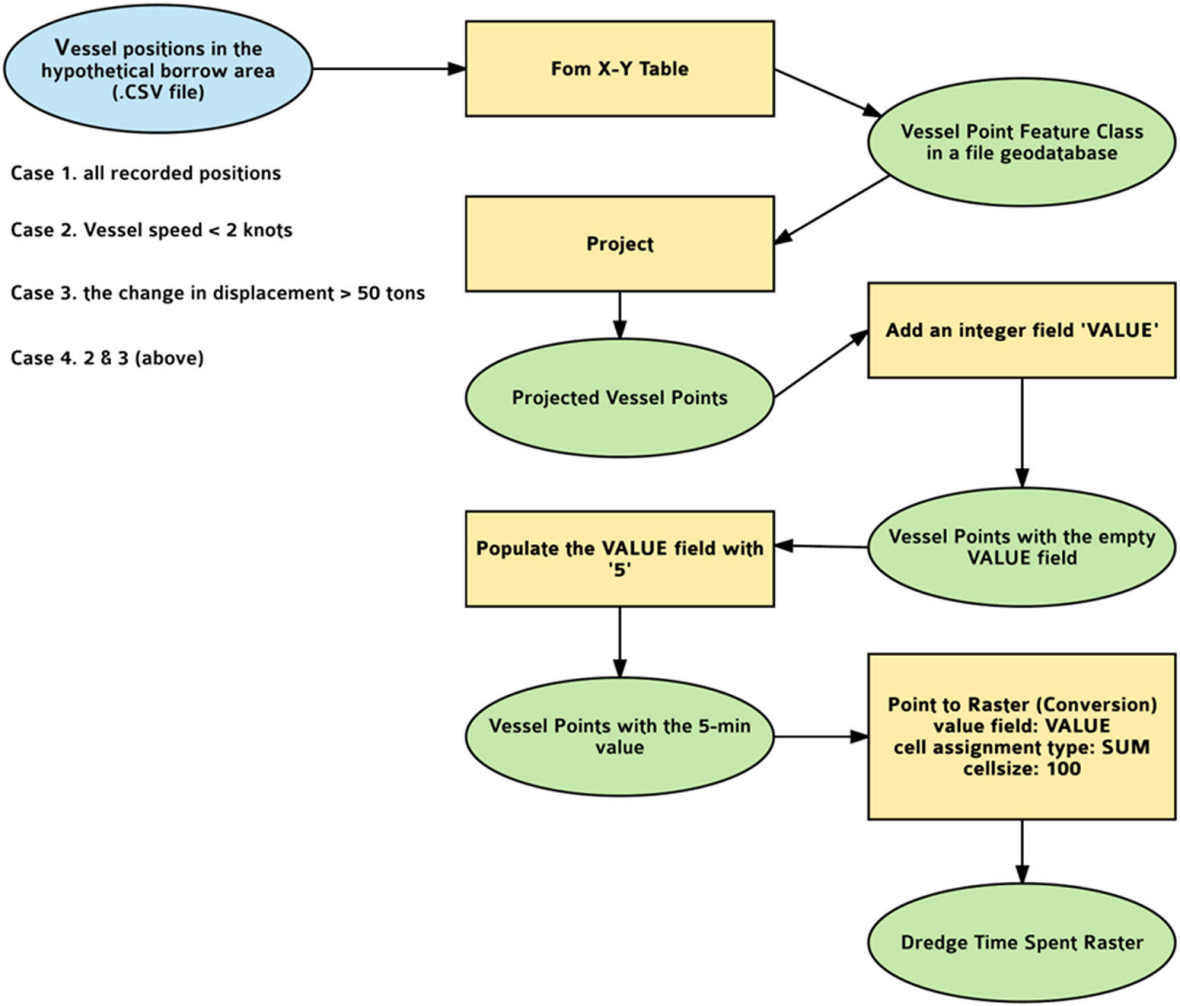
Model of the GIS procedure

This process (Fig. 2) was repeated four times, first using all locations in the designated area. Second, because dredgers travel at slower speeds when actually extracting sand, the speed may be used as a filter on the location data. Locations in the designated area when the vessel was traveling more slowly may discriminate between times of actual dredging and locations representing transit through the area. Because the data is a snapshot in a 5-min interval, the vessel could have been traveling more slowly anytime between the previous snapshot and the subsequent snapshot. As a result, the minimum speed for each entry was calculated over 15 min, around three successive entries. The dredging speed was taken as being less than two knots (Robert Ramsdell 2017, Great Lakes Dredge & Dock Company, personal communication). So a second heat map was generated on a subset of these locations when the vessel speed was less than two knots.

Third, by trial and error, the data in the designated borrow area were edited to include only positions where the change in displacement was greater than 50 tons. A fourth heat map was compiled from a subset of locations where the speed was less than two knots and displacement changed by more than 50 tons. The geospatial patterns and the summary statistics of the output raster within the hypothetical borrow area were examined for each case.

## Results

**Fig. 3:**
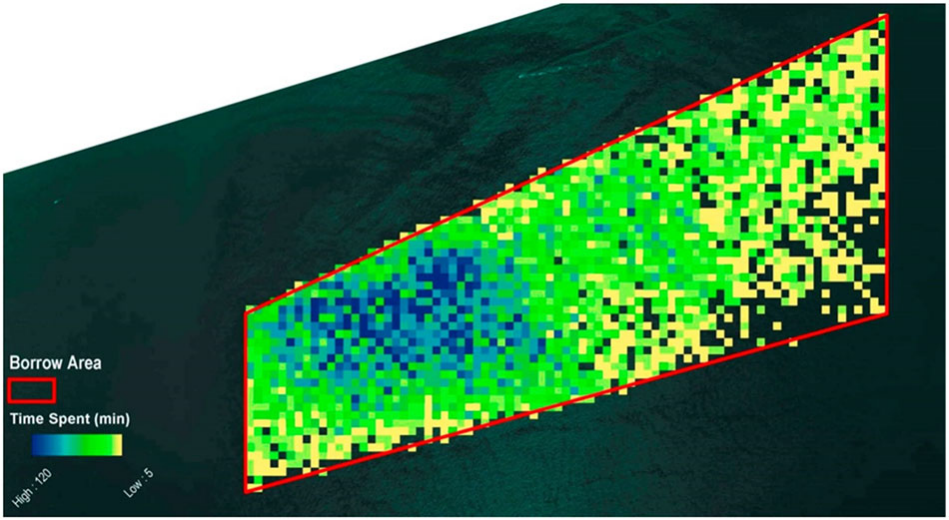
Occurrence of all dredge positions in the hypothetical borrow area

For this example, 48,484 individual locations were provided. Of those, 8732 positions, or 18% of the ship’s positions were in the hypothetical borrow area. Because each recorded position occupies a 5-min time period, the aggregate time the dredge spent in each cell can be calculated. The time the dredge spent in a single cell ranged from 1 to 120 min. The distribution was concentrated in the western section of the hypothetical borrow area (Fig. 3).

**Fig. 4:**
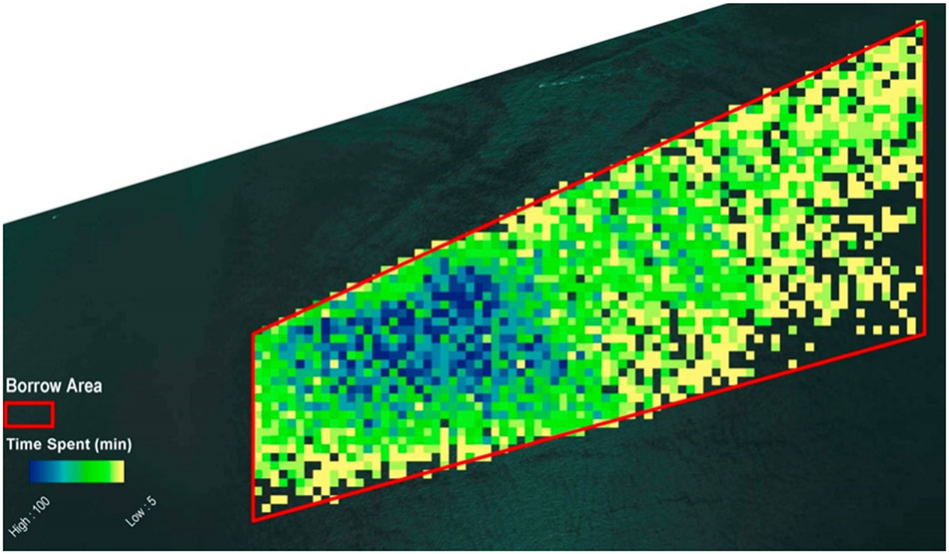
Occurrence of all dredge positions in the hypothetical borrow area when the speed was less than two knots

For a dredging speed less than two knots, 7729 positions were identified. The aggregate time that the dredge spent in a single cell ranged from 5 to 100 min. This distribution (Fig. 4) was very similar to that of all recorded positions (Fig. 3) with slow-speed activities concentrated in the western section of the hypothetical borrow area.

**Fig. 5:**
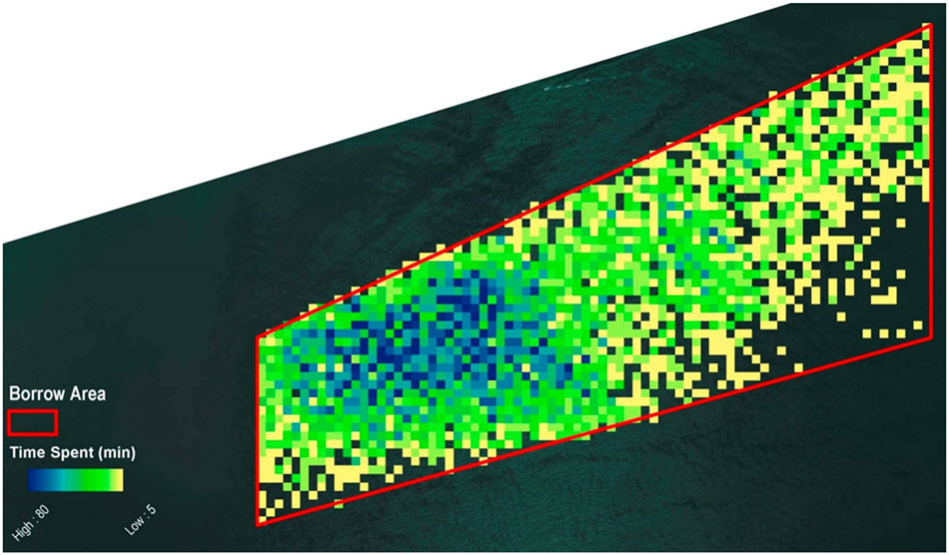
Occurrence of all dredge positions in the hypothetical borrow area showing an increase in displacement of more than 50 tons

**Fig. 6:**
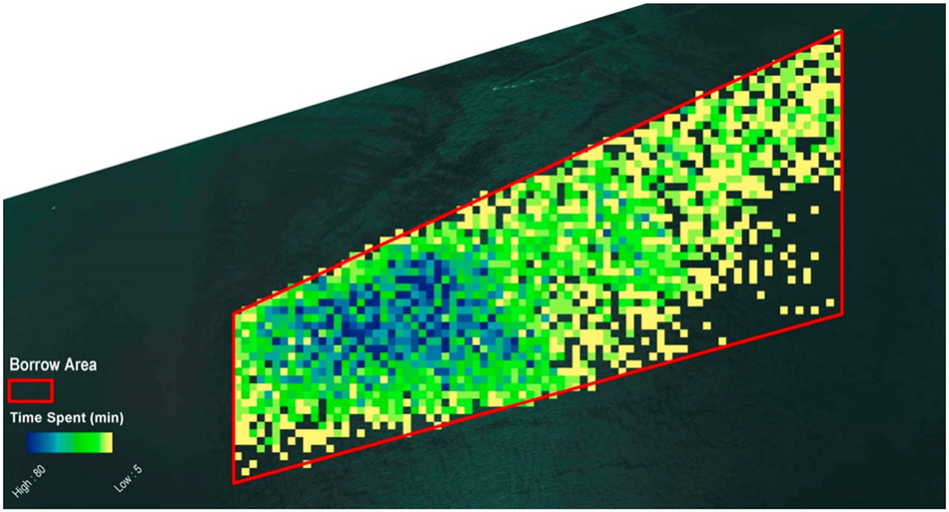
Occurrence of all dredge positions in the hypothetical borrow area showing an increase in displacement of more than 50 tons and a speed less than two knots

A total of 6371 positions recorded positive increases in displacement, indicating that the vessel was actively dredging (or possibly taking on water; Fig. 5). The total time spent in single cells ranged from 5 to 80 min. Applying both the filter for speed and that for increase in displacement, 6050 positions were identified. The aggregate time spent in a single cell ranged from 5 to 80 min (Fig. 6). In terms of the distribution of dredging intensity there did not seem to be a substantial advantage to selecting by speed or displacement. As a result, the basic EMS or AIS data would be adequate to capture dredging intensity.

It was suggested that a useful exercise would be a comparison of surface area disturbances estimated through dredging intensity analysis methods presented here, versus estimates obtained through pre-dredging and post-dredging bathymetry. Unfortunately, surveys for this area were not available. However, during this dredging operation, 98.5% of the vessel’s time was spent in a gridded area of 12.26 km^2^. The other 1.5% of the time was spent in a grid area of 7.89 km^2^ amounting to less than 10 min per cell. Although in such a short time it is unlikely that the entire 100 × 100 m grid would be covered, it also seems unlikely that a measurable difference in depth would be seen in that area so that, while the disturbed grid area was 20.15 km^2^, almost 40% might be undetected in difference maps pre-dredging and post-dredging

## Discussion

If the total volume of sand required for beach nourishment project is less than a few percent of total reserve of available offshore sand, this may be a long-term source of material. Otherwise, the physical replenishment of the sand blanket is one condition for sustainability of the resource. Replenishment depends on the rate at which sand may be redistributed by natural processes on the sea floor, but incomplete knowledge regarding sediment transport and the interactions between the shelf and nearshore system pose significant challenges in managing and possibly utilizing this resource. The inner continental-shelf is a mobile sea bed; however, determining net sediment fluxes remains an elusive goal. Rates of transport and regional patterns of pathways for mobile sand that would be needed to estimate sustainability of specific borrow areas are not well known. Modeling is possible (e.g., Warner et al. 2008; Byrnes et al. 2004) although the requisite data needed to exercise such models is often lacking.

Another approach relies on monitoring bedforms and changes in bathymetry tempered, as discussed earlier, by uncertainties in the process of bathymetric surveying. Repeated bathymetric surveys can document sand accumulation, if it is large enough. The storm conditions during Superstorm Sandy on the east coast of the U.S. shifted large sand deposits many yards and caused a foot or more thick layer of sand deposition (Goff et al. 2015). The rate of recovery of borrow areas might be documented by subsequent, post-dredging bathymetric surveys of the borrow areas. While dredging scars are known to persist for years, evidence of infilling of historical borrow areas was recognized after the borrow area was inactive for a couple of decades (e.g., Byrnes et al. 2004; Schwab et al. 2013). For example, evidence of infilling was recognized in offshore borrow sites along the US Atlantic coast (Schwab et al. 2013). One site excavated in 1997 was indistinct in 2012 perhaps indicating that ambient sand transport has substantially replenished the area. On the other hand, at another site dredged in 2009, up to two meters below the ambient sea floor, showed no evidence of morphological recovery after four years.

Although the number of benthic species in a borrow area might be reduced by more than 50% after dredging (Newell et al. 1998; Desprez 2000; Boyd and Rees 2003; Newell et al. 1998, 2004; ICES 2009, 2011; Krause et al. 2010), benthic communities can be sustained if they are resilient. Recovery of disturbed benthic communities to their original abundance, diversity and ecological function is probably best achieved if the physical environment is restored (Woods et al. 2016 Cooper et al. 2010. To judge the sustainability of ecosystem goods and services, estimates of dredging intensity might be overlain on resource maps to insure that the area of disturbed habitat is insignificant compared with the habitat’s total extent or that rare benthic habitats are minimally impacted. Dredging intensity appears to exert an influence on recovery rates of community structure that can range up to 15 years following dredging at high intensity (Turbeville and Marsh 1982; Boyd et al. 2003, 2004; Thrush et al. 2008; Birchenough et al. 2010; Wan Hussin et al. 2012; Waye-Barker et al. 2015).

Sustainable biological recovery can occur even if the physical setting persists in an altered state. Previous studies (e.g., De Backer et al. 2017) found the intensive dredging resulted in a change in substrate, which can ultimately cause a shift in the benthic community. Sediment may coarsen due to the exposure of coarser layers, or, on the other hand, the escape of the fine-grained fraction due to overflow could make the substrate more muddy. Depending on the intensity and frequency of natural disturbances and on the rate of natural sediment transport, such impacts could result in a shift in biological communities inhabiting the site and the creation of a more heterogenetic habitat overall (De Backer et al. 2017). While the resulting community structure may be different from the original, pre-dredging condition, it is possible that the new ecosystem still preserves its ecological function. Such transformations must be evaluated on a case-by-case basis in the framework of ecosystem-based management. Maps of dredging intensity might help guide these assessments.

## Conclusion

Clearly, the sustainable exploitation of offshore sand resources demands to be approached from multiple directions. The tactic described here offers one avenue. Monitoring data can be readily transformed into surrogates for dredging intensity at offshore borrow sites. It would seem that a time print heat-map gives a good view on the actual footprint of aggregate dredging at high resolution, even without filtering by vessel speed or changes in displacement, which might indicate active extraction. The procedure might be especially useful for assessing the extent of habitat disruption where the extraction of layers too thin to be detected in bathymetric surveys may still be impacting habitats. The interpretation of these data in terms of the recovery and sustainability of the resource still requires careful considerations using multiple, site specific factors. However, the strategy described here allows for comparison of usage between sites and for the targeting of subsequent environmental monitoring of site recovery.
